# The EU Midwifery Directive and Professional Strengthening in New EU Member States and Candidate Countries

**DOI:** 10.3390/healthcare14142224

**Published:** 2026-07-22

**Authors:** Lia Brigante, Daniela Drandić, Vanessa Vera, Ana Polona Mivšek, Tita Stanek Zidarič, Magdalena Kurbanović, Lenka Veselá, Radka Wilhelmová, Małgorzata Nagórska, Daniela Lyutakova, Alina Liepinaitienė, Blerta Kryeziu, Sevil Hakimi, Vedran Đido, Larysa Cheprasova, Olha Honcharenko

**Affiliations:** 1 International Confederation of Midwives (ICM), 2514 AE The Hague, The Netherlands; lia.brigante@rcm.org.uk (L.B.); d.drandic@internationalmidwives.org (D.D.); 2 Faculty of Health Sciences, University of Ljubljana, 1000 Ljubljana, Slovenia; polona.mivsek@zf.uni-lj.si (A.P.M.); tita.zidaric@zf.uni-lj.si (T.S.Z.); 3 Faculty of Health Studies, University of Rijeka, 51000 Rijeka, Croatia; magdalena.kurbanovic@uniri.hr; 4 Department of Health Sciences, Faculty of Medicine, Masaryk University, 625 00 Brno, Czech Republic; lenka.vesela@med.muni.cz (L.V.); rwilhelm@med.muni.cz (R.W.); 5 Institute of Nursing, Faculty of Health Sciences and Psychology, Collegium Medicum, University of Rzeszów, 35-310 Rzeszów, Poland; mnagorska@ur.edu.pl; 6 International Board of Certified Lactation Consultants, 2500 Baden, Austria; lyutakova.midwife@gmail.com; 7 Department of Environmental Sciences, Faculty of Natural Sciences, Vytautas Magnus University, 44248 Kaunas, Lithuania; alina.liepinaitiene@smk.lt; 8 Faculty of Medicine, University of Prishtina, 10 000 Prishtina, Kosovo; blerta.kryeziu@uni-pr.edu; 9 Faculty of Health Sciences, Ege University, İzmir 35575, Turkey; sevil.hakimi@ege.edu.tr; 10 Faculty of Health Studies, University of Sarajevo, 71000 Sarajevo, Bosnia and Herzegovina; vedran.djido@fzs.unsa.ba; 11 Ukrainian Midwives’ Union, 04211 Kyiv, Ukraine; lcheprasovaukrmidwives@gmail.com; 12 Ukrainian Association of Midwives, 04210 Kyiv, Ukraine; gorinivna@gmail.com

**Keywords:** midwifery, European Union directive, midwifery education, professional strengthening, EU directive on minimum professional qualifications, women’s health, EU accession, professional regulation

## Abstract

This article examines the impact of the EU Directive on the recognition of professional qualifications (2005/36/EC) on the development and strengthening of midwifery in countries that joined the European Union in recent decades, with a particular focus on Central and Eastern Europe. Focusing on experiences from Croatia, Poland, Slovenia, Bulgaria, and the Czech Republic, the paper explores how alignment with the Directive influenced education reform, professional recognition, and scope of practice. Drawing on the experiences presented, we argue that the Directive has functioned as more than a technical requirement for EU accession. By establishing minimum educational standards, it supported the transition from vocational to university-level education, facilitated pathways for the existing workforce to align with new qualification requirements, and contributed to the recognition of midwifery as a distinct regulated profession. It also provided a clear framework for defining midwives’ competencies and scope of practice within health systems, acting both as a protective mechanism, by safeguarding professional standards, autonomy, and professional identity, and as an enabling framework by supporting workforce development, educational reform, and the integration of midwives into health systems during periods of institutional transition. The lessons drawn from these national experiences can inform the ongoing update of the Directive, led by the EU Commission, by illustrating how legislative alignment can strengthen midwifery education and professional recognition, while also supporting countries undergoing or preparing for EU accession.

## 1. Introduction

Joining the European Union (EU), a political and economic union currently comprising 27 Member States, is a long and demanding process. Countries that want to become members have to meet a set of criteria and align their national laws and systems with EU frameworks. Since the major enlargement rounds of 2004, 2007 and 2013, thirteen countries from Central, Eastern and South-Eastern Europe have joined the EU, including Poland, the Czech Republic, Slovenia, Lithuania, Bulgaria and Croatia. At the same time, several countries, including Bosnia and Herzegovina, Kosovo, Turkey and Ukraine, are currently candidates or potential candidates for accession and are progressively aligning their systems with EU legislation as part of this process. One component of this EU legislative framework is the Directive on Minimum Professional Qualifications (2005/36/EC), which sets the minimum training requirements and standards for education for seven regulated professions, including midwives [1]. EU accession and enlargement is often analysed in terms of economic, legal and political reform or perspectives, giving less attention to what this process means for health professions and their regulatory frameworks.

This article looks at what happened to midwifery in countries that joined the EU in recent decades, particularly in Central and Eastern Europe, while also incorporating perspectives from countries that are currently candidates or potential candidates for accession. The analysis is informed by the experiences of authors from Croatia, Poland, Slovenia, Bulgaria, the Czech Republic and Lithuania, complemented by reflections from Bosnia and Herzegovina, Kosovo, Türkiye and Ukraine, where preparations for alignment are already underway.

We argue that, apart from establishing minimum educational standards, the Directive has functioned as both a protective and an enabling framework for the profession. By protective, we refer to the way in which it provided an external policy benchmark that supported reforms in education, regulation and professional recognition, helping safeguard the distinct identity and role of midwifery during periods of transition. By enabling, we refer to its role in facilitating broader reforms, including the transition to university-level education, clearer scope of practice and stronger professional regulation. In doing so, the paper contributes to a largely overlooked dimension of EU enlargement: its impact on the strengthening of health professions.

## 2. The Directive as a Protective Tool

### 2.1. Education Reform

Since the Directive establishes education and training requirements for midwives, reform of midwifery education was one of the biggest and most visible changes. To join the EU, countries had to adapt their education systems related to midwifery to align with EU standards. This led to significant changes in the way midwives were educated.

In most of the countries that joined the EU during the 2004–2013 enlargement rounds, including Croatia, Poland, Bulgaria, the Czech Republic and Lithuania, prior to the accession process, training for midwives was limited exclusively to the secondary or vocational level. This meant that it was not part of the higher education system, and midwives did not have access to academic-level education. Additionally, in some countries, like Poland and Croatia, higher education for midwives was available only through a nursing degree with a gynaecological–obstetric specialization [2,3,4]. EU accession prompted these countries to move toward university-level education, introducing bachelor’s degrees for the first time.

For example, in the Czech Republic, midwifery education transitioned from a two-year programme to a three-year bachelor’s degree, accompanied by major curricular reforms and the introduction of student-centred learning and mentoring systems. In Poland, accession accelerated the transfer of midwifery education entirely into the higher education sector, leading to the establishment of bachelor’s and master’s degree programmes. In Croatia, the introduction of bachelor’s-level midwifery education subsequently paved the way for the establishment of a master’s programme, which would have been difficult to achieve without this earlier alignment [2].

Transitions for midwives who had already completed their education under the previous system in secondary and vocational educational programmes also took place, since they faced the challenge of having qualifications that no longer met the Directive’s standard of a bachelor’s-level degree. As a result, bridging programmes were introduced, which provided practicing midwives with a pathway to upgrade their education to a higher level and obtain a diploma recognised in other EU Member States [3]. This ensured that midwives educated in the previous system were not left behind and could benefit from improved educational opportunities during the transition period associated with accession. However, these bridging programmes should have, by definition, been a temporary measure, which has not been the case in all countries.

Beyond the level of education, the content of the programmes also changed. Curricula were redesigned to reflect midwifery competencies, and there was a significant increase in the number of hours dedicated to both theoretical learning and clinical education. This meant an expansion of time that students spent working directly with women and families in real care settings [5]. For example, in Slovenia and the Czech Republic, clinical placements expanded beyond hospitals to include primary care, maternity services and community care centres. This led to a broader understanding of what midwifery is, the settings in which it is practised, and it also strengthened students’ preparation for autonomous practice.

Another important impact of this period of transition was the role of midwives themselves in education. As programmes were established and expanded, midwife educators were increasingly required. In some countries, like Croatia, the creation and development of postgraduate programmes also created a new pathway for midwives to progress their career into teaching and research.

All these changes to the education and training system have had lasting effects on the professional development and identity of midwives.

### 2.2. Professional Recognition and Autonomy

One of the clearest effects of the accession process on midwifery in these countries has been how the profession is defined and recognised. In many of the countries that joined the EU, midwifery had struggled to be recognised as a distinct profession separate from nursing, and midwives’ autonomy was consistently contested, especially because of the growing medicalisation of childbirth [6]. In addition, midwifery had also been closely linked and, in many cases, conflated with nursing. For example, in Slovenia and Croatia, midwifery had historically been recognised within healthcare systems but remained institutionally linked to nursing and strongly influenced by obstetric-led models of care. Both professions shared the same regulatory frameworks, and in some cases the same education pathways [7,8,9,10]. Similarly, in Poland, although midwifery was recognised as a profession, educational and regulatory reforms were still required to consolidate its status as an autonomous health profession. The EU accession process brought these issues to the forefront.

Aligning with the Directive meant that countries had to clarify what midwifery was, and gave a concrete impetus to address these issues. In that context, midwifery began to be separated from nursing to have its own regulation and in education systems. This included making a distinction between midwifery and nursing education, establishing regulatory bodies, and adopting legislation, such as midwifery acts, that formally recognised the profession [11]. In Croatia, this process resulted in the establishment of the Croatian Chamber of Midwives and the adoption of a dedicated Midwifery Act, providing a legal framework that formally recognised midwifery as a distinct regulated profession.

Additionally, because midwives were now responsible for teaching midwifery students, new generations of students were educated by educators who helped to transmit a distinct professional identity and philosophy of care.

Another aspect was the development of professional standards such as codes of conduct and ethics. In Slovenia, the European Commission specifically recommended the development of a separate code of ethics for midwives and greater visibility of midwives within professional organisations [12]. In several countries, this work was undertaken by professional associations that were newly formed, which formalised what it meant to practise as a midwife in technical terms, but also in terms of values and responsibilities. This was an additional step in reinforcing midwifery as a regulated and legitimate profession within the health system.

### 2.3. Clarifying Scope of Practice

A more concrete and operational scope of midwifery practice was another effect of the accession process for the profession. The Directive provided countries with a reference point for organising midwifery care and offered a clearer definition of what midwives actually do. For example, in the Czech Republic, recommendations made during the accession process called for midwives to independently manage low-risk pregnancies and births, expand services into primary care, and provide continuity of care. Similarly, in Croatia, the Directive informed revisions to national legislation defining midwives’ competencies across pregnancy, birth and the postnatal period. In contexts where midwifery competencies had been vague or blurred, either because they overlapped with nursing or because autonomous practices did not take place, this was particularly important. The changes in education and regulation were key in establishing an applicable and practical definition of the profession.

The competencies defined in the Directive include providing care during normal pregnancy, conducting spontaneous births, caring for women and newborns throughout the postnatal period, and identifying complications requiring referral. During accession, these competencies became an important benchmark for revising national legislation and defining the responsibilities of midwives within health systems. In Poland, these reforms were reflected in legislation that expanded midwives’ competencies to include prescribing selected medicines or referring women for specific diagnostic tests. These legislative changes aligned professional responsibilities more closely with the competencies acquired through university education.

While there is still work to be done in translating these changes into everyday practice, the clarification of what midwives are educated, qualified, and authorised to do has helped strengthen the understanding of their role within health systems, reinforcing their contribution to sexual, reproductive, maternal, newborn and adolescent health services.

Alongside the experiences of countries that have already joined the EU, similar patterns can be seen in countries that are currently candidates for accession. Before they are formally required to align, the Directive is being used as a reference point, helping countries prepare for that process and shaping changes in education, regulation, and professional recognition. The prospect of joining the EU encourages countries to begin this alignment early. In this way, the Directive does not only apply after accession but also plays a protective and guiding role beforehand.

Overall, what these experiences show, across different countries and contexts, is that the Directive, for these countries joining the EU, functioned as a form of protection for the profession during and after this period of transition. By setting the minimum educational requirements for midwives, it required a shift from secondary-level training to university-level programmes, alongside a clearer scope of practice and formal professional recognition. This gave midwives and the profession something concrete to stand on. The Directive provided a protective foundation for the profession, enabling reforms that, in many cases, would have been difficult to achieve.

## 3. Discussion

The experiences presented in this paper suggest that the role of the Directive extended beyond establishing minimum educational requirements for automatic recognition of professional qualifications. Across different country contexts, it provided an external policy benchmark that supported reforms in education, regulation and professional recognition, particularly during periods of significant institutional transition associated with EU accession. Rather than acting solely as a technical requirement, the Directive offered governments a common framework against which national legislation and educational systems could be modernised.

In this sense, minimum standards functioned not as a constraint, but as a catalyst for professional development, supporting competency-based education, strengthening the recognition of midwifery as a distinct profession, and providing greater clarity regarding the role and competencies of midwives within health systems. Instead of limiting national health and education systems, these standards enabled the development of a stronger professional framework.

Minimum standards gave countries both direction and justification for reform. They helped align education systems with competency-based approaches, supported the recognition of midwifery as a distinct profession and clarified the role and competencies of midwives within health systems.

These lessons matter for future EU accession processes and for countries continuing to align with EU standards. EU-level minimum standards serve as both a tool and driver for professional development. At the same time, by strengthening midwifery, they contribute to improving health systems, including the quality of care for women and newborns. In this way, the Directive supports the development of a workforce capable of delivering improved health outcomes.

These findings may also have broader relevance beyond the European Union. The experience of recently acceded countries demonstrates how regional minimum standards can contribute to strengthening health professions by supporting education reform, professional regulation, and clearer and broader scopes of practice. This is consistent with the broader vision reflected in the ICM Global Standards for Midwifery Education and the ICM Essential Competencies for Midwifery Practice [13,14], which similarly seek to promote high-quality, competency-based midwifery education and practice internationally. Although the mechanisms differ across regions, the experiences described here suggest that shared professional standards can serve as important drivers of workforce development and quality of care.

This perspective is based on the experiences of selected countries represented by the contributing authors and is therefore not intended to provide a comprehensive assessment of all EU Member States or accession countries. Nevertheless, the consistency of the experiences across different national contexts highlights an important and often overlooked dimension of EU enlargement. These lessons are particularly relevant as the Directive undergoes revision, providing an opportunity to build on the progress achieved over the past two decades and ensure continued alignment with current evidence and international standards, thereby strengthening the midwifery profession and supporting high-quality care for women and newborns across Europe.

## 4. Conclusions

The experiences presented in this paper suggest that, for countries undergoing EU accession and alignment, the Directive has functioned as more than a technical requirement for the recognition of professional qualifications. It has provided a common framework that supported reforms in midwifery education, professional recognition and scope of practice, helping strengthen the profession during periods of important transition. While the extent of implementation has varied across countries, these experiences demonstrate the important role that regional minimum standards can play in supporting professional development and health system strengthening.

As the Directive is currently being updated, this evidence presents an important opportunity to build on these gains and ensure alignment with current evidence and international standards, including the ICM Global Standards for Midwifery Education and the Essential Competencies for Midwifery Practice [13,14]. In this way, the Directive will continue to support the profession and to ensure the delivery of high-quality care for women and newborns across Europe.

## Data Availability

No new data were created or analyzed in this study. Data sharing is not applicable to this article.

## Abbreviations

The following abbreviations are used in this manuscript:

EU	European Union
ICM	International Confederation of Midwives
